# Homes became the “everything space” during COVID-19: impact of changes to the home environment on children’s physical activity and sitting

**DOI:** 10.1186/s12966-022-01346-5

**Published:** 2022-10-21

**Authors:** Michael P. R. Sheldrick, Nils J. Swindell, Amie B. Richards, Stuart J. Fairclough, Gareth Stratton

**Affiliations:** 1 Research Centre in Applied Sports, Technology, Exercise and Medicine (A-STEM), Swansea University, Swansea, SA1 8EN UK; 2 Movement Behaviours, Health and Wellbeing Research Group, Department of Sport & Physical Activity, Edge Hill University, St Helens Road, Ormskirk, L39 4QP Lancashire UK

**Keywords:** Sedentary behaviour, Family, Home-based, Pandemic, House, Accelerometers, Youth

## Abstract

**Background:**

During the 2020 UK COVID-19 lockdown restrictions, children spent almost all of their time at home, which had a significant influence on their physical activity (PA) and sedentary behaviour. This study aimed to: 1) determine changes to the social and physical environment at home and children’s home-based sitting, PA, standing and sitting breaks as a result of the COVID-19 restrictions; and 2) examine associations between changes at home and children’s movement behaviours.

**Methods:**

One hundred and two children had their PA and sitting, standing and sitting breaks at home objectively measured pre-COVID-19 and during the first COVID-19 lockdown (June-July 2020). Children’s parents (*n* = 101) completed an audit of their home physical environment and a survey on the home social environment at both time points. Changes in the home physical and social environment and behavioural outcomes were assessed using Wilcoxon signed ranked tests, paired t-tests, or chi-square. Repeated linear regression analyses examined associations between changes in homes and changes in the home-based behavioural outcomes.

**Results:**

During COVID-19, households increased the amount of seated furniture and electronic media equipment at home. The number of books and PA equipment decreased and fewer parents enforced a screen-time rule. Children’s preference for physical activities and socialising at home decreased. Time at home and sitting at home increased during COVID-19, whilst PA, standing and sitting breaks decreased. Both MVPA and TPA were positively associated with child preference for PA, and negatively associated with attending school. Sitting was negatively associated with child preference for PA and child preference for socialising at home. Media equipment was negatively associated with sitting breaks, whilst PA equipment was positively associated with standing.

**Conclusion:**

The COVID-19 restrictions forced children to spend almost all their time at home. Children’s PA, standing, and sitting breaks at home declined during the restrictions, while sitting increased. Mostly negative changes occurred in homes, some of which impacted children’s behaviours at home. To avoid the changes persisting post-lockdown, interventions are needed to reset and promote children’s PA and discourage prolonged sitting time.

**Supplementary Information:**

The online version contains supplementary material available at 10.1186/s12966-022-01346-5.

## Introduction

Regular physical activity (PA) is vital for children’s physical and psychological health [1]. On the other hand, sedentary behaviours, particularly some screen-based, are associated with unfavourable body composition, metabolic profiles, and mental health outcomes [2]. Considering that children spent most of their time at home even before the pandemic [3, 4], and ecological models posit that behaviour is most likely influenced by the environment in which it occurs [5], the home is an important sphere of influence on children’s PA and sedentary behaviour [6, 7]. Parents are particularly influential in the home [6]. Indeed, parental PA, support and co-participation are strong correlates of children’s PA [7–9]. Sedentary parents are likely to have sedentary children, and parental limits on children’s screen-time have been associated with less sedentary behaviour in children [6, 10]. The physical environment at home also has an importance influence. Media equipment availability has been positively associated with sedentary time [7], while musical instruments are negatively related [7]. Conversely, PA equipment is positively associated with PA [11, 12] and inversely related to sedentary time [6, 11], albeit not at home [7, 11].

On March 11, 2020, the COVID-19 (Coronavirus) outbreak was declared a global pandemic by the World Health Organisation [13]. On March 23^rd^ 2020, the United Kingdom (UK) government announced a national lockdown and ordered the entire population to “stay at home” to mitigate the spread of the virus. Members of the public were only permitted to leave their homes for food, medical supplies and for a short bout (60 min) of local daily exercise [14]. Children no longer had access to school-based physical activities such as physical education and break time and walking to/from school. Team sports leagues and fitness and activity classes such as dance, martial arts and gymnastics were also cancelled or postponed. Such measures profoundly limited children’s opportunities to be physically active, and increased sedentary time as a result [15, 16]. In addition, the physical distancing/isolation measures and resultant stressors may have negative short- and long term mental health consequences, including anxiety, depression and behavioural problems [17]. The combination of inactivity and social distancing/isolation measures could have a synergistic effect on children’s lifestyle behaviours and their mental health and wellbeing [18]. Investigating the impact of the pandemic on children’s PA and sedentary time during COVID-19 is essential, not only to prevent the inactive behaviours from becoming permanent, but also to support children as they continue to experience pandemic-related stressors.

Several studies report that children were less active and engaged in increased screen-time at home during COVID-19 [16, 19, 20]. A small number of studies have investigated the correlates of the decline in PA and increase in sedentary behaviour [21, 22], yet few studies have explored environmental correlates [15, 23]. Indeed, ecological models posit that the environment has an important influence on children’s PA and sedentary behaviour [24, 25]. During COVID-19, homes became the “everything space”, places for play, work, school, eating and socialising, which led to significant changes in daily life at home for children and their families [26, 27]. We hypothesised that these enforced changes would impact on the physical and social home environment and in turn children’s PA and sedentary behaviour during COVID-19. To the authors’ knowledge no study has investigated the activity-related changes in homes during COVID-19 and their impact on children’s PA and sedentary behaviour at home. Further, studies using device-based measures of children’s PA and sedentary behaviour pre and during COVID-19 are scarce [15, 23].

The UK-HomeSPACE study measured children’s PA and SB, and environmental factors in the home prior to COVID-19 [7, 28], allowing a unique opportunity to repeat these measures on the same cohort of children during the pandemic. The aims of the HomeSPACE-COVID-19 study were to: 1) identify changes in the social and physical environment at home and children’s home-based sitting, PA, standing and sitting breaks in response to the COVID-19 pandemic; and 2) examine the impact of changes at home on children’s home-based sitting, PA, standing and sitting breaks.

## Materials and methods

### Participants and settings

Two hundred and thirteen children and their parents participated in the HomeSPACE-UK project between November 2017 and July 2018. Of the 213 children, 102 children (49% response rate) and their parents (*n* = 101) agreed to take part in the HomeSPACE-COVID-19 follow up study during the first COVID-19 lockdown (June-July 2020). As a thank you for participating, families were given a £20 Amazon voucher and personalised feedback reports from the follow up study. The protocols for the pre-COVID-19 (REC: 2016–110) and during COVID-19 (REC: MS_2020-029a) studies were approved by the Swansea University ethics committee. A target sample of 96 participants was set based on cross-sectional data from the same sample in which multiple regression generated R^2^ values of 0.21 to 0.25 for home based sitting, standing, sitting breaks, MVPA and total PA (TPA) after controlling for covariates [7]. Using the R package *pwr* and targeting 80% power and alpha of 0.05, a sample of size of 87 was required to run a general linear model [29]. The target sample size was increased by 10% (*n* = 96) to account for data attrition.

### Physical environment of the home

The validated HomeSPACE-II instrument [30] was used to assess the home physical environment in relation to children’s PA and sedentary behaviour both pre-COVID-19 and during the first COVID-19 lockdown. Parents were asked to walk around their house and garden and complete an audit of the presence, amount, and accessibility of 41 media, musical, PA and seated furniture items for each area of the house and garden. For each item, accessibility was rated on a scale of (A) ‘put away and difficult to get to’ to (D) ‘in plain view and easy to get to’. In addition, there were questions relating to electronic media (smartphones, TV service, movie/TV streaming service, DVDs) and equipment that could not be captured in the audit (books and fitness trackers). The number of individual items were summed for PA equipment, musical instruments, media equipment and seated furniture. Lastly, summary scores (reflecting availability and accessibility) were created for each item category by multiplying each item by their accessibility score (A = 1; B = 2; C = 3; D = 4). The higher the summary score the greater overall “presence” the item has in the home. All items used in this study have strong test–retest reliability (ICC =  > 0.80, K =  > 60) [31, 32] and criterion validity (r =  > 0.75) [30].

### Home-based PA, sitting and sitting breaks

Before (2017–18) and during the first COVID-19 lockdown, total and moderate-vigorous physical activity (TPA and MVPA) and postural behaviours (i.e., sitting and sitting breaks) were assessed with the ActiGraph GT9X (Pensacola, Florida, USA) and the activPAL3 micro (PAL Technologies, Glasgow, UK) accelerometers, respectively. Pre-COVID-19, monitors were fitted at schools to ensure correct attachment. This was not feasible during lockdown, because most children were not attending school at the time of data collection. Instead, the monitors were delivered to families’ homes with instructions on correct attachment. Children were asked to wear the monitors continuously (including bathing, but not swimming) for seven consecutive days pre-COVID-19 and eight consecutive days during the first lockdown to allow for possible delays in attaching the monitors. Parents completed a diary, recording when the child was at home [7], asleep, illness days, and periods when the device was removed.

The activPAL has previously been validated in children [33]. Briefly, activPAL data were downloaded using the manufacturer software (V8.10.8.32, PAL technologies, Glasgow, UK) and the subsequent event.csv files were processed in PAL-V1.1 (Leicester, UK) with a validated algorithm that identified waking hours, prolonged non-wear time (≥ 5 h) and invalid data [34, 35]. Diary-reported non-wear periods deemed plausible were removed. Additionally, sitting/lying or standing bouts lasting ≥ 3 h with no transitions were also classified as non-wear and removed [36].

The ActiGraph GT9X was placed on the child’s non-dominant wrist [37]. The device data was collected at a 30 Hz sampling rate [38] and summed over 5-s epochs. Files were initialised, downloaded and processed using ActiGraph software (ActiLife V6.13.3). Wrist-worn vector-magnitude cut-points [39] were utilised, whereby TPA and MVPA were categorised as ≥ 306 and ≥ 818 counts/5 secs, respectively. An algorithm was used to identify non-wear time (≥ 90 consecutive minutes of zero counts) [40]. Parent-reported time at home, imported into the ActiLife V6.13.3 and processing PAL software, were paired with time-stamped data, allowing time spent in PA and postural behaviours at home to be estimated, respectively. To be included in the analyses, children needed at least 3 days with ≥ 3 h of data at home when the device was worn for ≥ 75% of the time [41]. Minutes in total physical activity (TPA) and MVPA and postural behaviours were divided by wear time at home and multiplied by 60, constituting the dependent variables as averages/hr [42]. A more detailed explanation of the activity monitoring protocol and data processing techniques can be found elsewhere [7].

### Children’s demographic and anthropometric measures

Pre-COVID-19, children had their stature and body mass measured by trained researchers at their respective schools using a portable stadiometer (Seca 213, Hamburg, Germany) and electronic weighing scales (Seca 876, Hamburg, Germany), respectively, using standardised procedures [43]. During the first lockdown, this protocol was not feasible due to social distancing measures, therefore parents were given directions on measuring stature and body mass using a tape measure and standard bathroom scales, respectively [43]. Indeed, Parent-report reported height and weight has reasonable validity when compared with objective measurements [44, 45]. Body mass index (BMI) z-scores, were calculated using World Health Organisation (WHO) growth reference data [46].

### Additional measures

Parents reported their child’s age, sex, whether they owned or rented their home, education status (some secondary school/ completed secondary school/trade qualifications or apprenticeship/diploma or certificate/ university degree or higher), family situation (single parent/two parent/other), annual household income before tax, home postcode, whether they owned a dog, and the number of children living at home. Season of measurement included: Spring (March–May), Summer (June–August), Autumn (September–November) and Winter (December-February) were also recorded. The Welsh Index of Multiple Deprivation (WIMD), calculated from postcodes, was used as an indicator of socioeconomic status (SES) [47]. The WIMD scores take into account eight domains of area deprivation; employment; health; income; housing; community safety; access to services; education; the environment. For descriptive purposes, SES was stratified into tertiles according to WIMD scores; low (1–636), medium (636–1272) and high (1272–1909).

### Family social and individual factors

Items from the HomeSPACE-I instrument were used to assess parental and child perceptions, preferences and priorities within the home space [48]. All items in the present study have been shown to exhibit at least acceptable reliability (ICC =  > 55) [31], and internal consistency (α =  > 0.55) [49, 48]. The following social and individual factors were assessed: importance of children’s activity at home (8 items), importance of home features (8 items) and equipment (13 items); child (7 items) and parent (7 items) activity preferences at home; and child social preferences (2 items). Parents’ perceptions of space for play, safety and connection between areas (16 items) and the presence of rules relating to outdoor safety, media and indoor play at home were also assessed. These items are described in full elsewhere [48]. The outdoor safety rules score summed “yes” responses on three rules (yes/no): “Stay close/within sight of house/parent,” “do not go into street,” “do not ride bike on street” [50]. The media rules score summed “yes” responses on three rules: “no screen-use before homework”, “a maximum number of hours per day of screen-use” and “no screen-use at the dinner table” [51]. Lastly, the indoor rules score summed “yes” responses on two rules: “no running in the house” and “no ball games in the house” [52, 53].

### Factors relating to home life during COVID-19

Six questions were included to capture factors created by the pandemic. Firstly, parents were asked if the child/children taking part in this study were attending school: no, they are at home; yes, most days of the week; yes, sometimes; and yes, but a different school. Parents were also asked if their children were being home schooled (yes/no) and how the day was structured around home-schooling (five categories ranging from “very structured” to “not at all structured”). The parents completing the questions were also asked if they were working from home (four categories ranging from “yes, a full day every day” to “not at all”), whether both parents were at home (yes/no), and if the other parent was at home whether they were working from home (5 categories ranging from “yes, a full day every day” to “not at all”. See instruments provided as supplementary files.

### Statistical analysis

Complete ActivPAL, ActiGraph, physical and social environment data were received at both time points for 88 (85%), 90 (87%), 101 (99%), and 102 (100%) children, respectively. Cases with missing data were deleted listwise. The data are presented as means and standard deviations (SD) or absolute and relative prevalence (*n*, %) for categorial variables, unless stated otherwise. Differences in key baseline characteristics between families that only participated pre-COVID-19 and those who participated both pre-COVID-19 and during COVID-19 were explored by independent t-test (continuous variables) or chi-square test (χ^2^) [categorial variables]. For families that participated pre-COVID-19 and during COVID-19, the average change from baseline to follow up was also calculated. Differences in home environment parameters at baseline and follow up were evaluated using Wilcoxon signed Rank test, paired t-test, or chi-square.

To examine the associations between changes in the five home-based behavioural outcomes, and the home environment, change scores between pre-COVID-19 and during the first lockdown were calculated for both the predictor and dependent variables that showed significant change (Tables 1 and 2).

**Table 1:** Descriptive statistics and differences for the social and physical environmental variables between pre-COVID-19 and during COVID-19

	**Pre-COVID-19 (2017–2018)**	**During COVID-19 (2020)**	***P***
**Physical environment (*n* = 102)**			
** Audit variables, m (SD)**			
Number of PA equipment items	30 (18.4)	28.9 (16.5)	0.02*
PA equipment accessibility and availability score ^a^	94 (66.6)	86 (50.2)	0.01*
Number of seated furniture items	20.7 (7.9)	22.2 (8.5)	0.01*
Seated furniture accessibility and availability score ^a^	81.4 (30.1)	86.5 (33.1)	0.01*
Number of media equipment items	11.8 (4.7)	13 (4.8)	0.01*
Media equipment accessibility and availability score ^a^	45.2 (18.7)	47.7 (18.4)	0.15
Number of bedroom media equipment items	1.7 (1.6)	2.2 (1.9)	< 0.01*
Bedroom media equipment accessibility and availability score ^a^	6.2 (5.8)	7.9 (6.9)	< 0.01*
Number of musical instrument items	2.3 (2.1)	2.4 (2.7)	0.39
Musical instrument accessibility and availability score ^a^	7 (7.1)	8.3 (9.3)	0.33
** Electronic media equipment**			
TV service, %			
Digital (e.g., SKY, BT etc.)	77%	82%	0.08
Freeview or other	23%	18%	0.08
Movie/TV streaming (e.g., Netflix, Amazon TV etc.) [% yes]	75%	92%	< 0.01*
Number of DVDS	3.7 (1.5)	3.7 (1.5)	0.13
Number of smartphones (mode)	3 (0.8)	3.3 (0.6)	< 0.01*
** Other equipment, m (SD)**			
Number of books	4.3 (1.5)	4 (1.4)	< 0.01*
Number of active video games (e.g., Wii sports, Just dance etc.)	2 (0.7)	1.9 (0.7)	0.46
Number of fitness trackers (e.g., fitbit, Garmin etc..)	2 (0.8)	2.4 (0.8)	< 0.01*
**Social environment (*n* = 102)**			
** Preferences, m (SD)**			
Child activity preferences at home ^2^	3.3 (0.8)	3 (0.9)	< 0.01*
Child social preferences at home ^2^	3.7 (1)	3 (0.9)	< 0.01*
Parent activity preferences at home ^2^	3.2 (0.6)	3.3 (0.6)	0.17
** Parental perceptions of the home physical environment, m (SD)**			
Outdoor space for play ^1^	7.5 (2.2)	7.2 (2.3)	0.08
Indoor space for play ^1^	10.2 (1.7)	9.9 (1.8)	0.08
Back outdoor supportiveness ^1^	10.9 (1.9)	11.2 (1.4)	0.20
Front outdoor visibility and connection ^1^	6.2 (3.3)	6.3 (3.5)	0.80
Front outdoor safety and access ^1^	7.2 (2.9)	7.5 (3)	0.18
** Activity priorities, m (SD)**			
Parent perceived importance of engaging in active play at home ^3^	4.2 (1.3)	4.2 (0.6)	0.46
Parent perceived importance of using electronic media equipment at home ^3^	2.2 (0.7)	2.2 (0.7)	0.95
** Home feature priorities, m (SD)**			
Parent perceived importance of the indoor living space at home ^3^	2.4 (0.8)	2.3 (0.7)	0.14
Parent perceived importance of space for play at home ^3^	4.2 (0.7)	4.3 (0.6)	0.22
** Home equipment priorities, m (SD)**			
Parent perceived importance of electronic media equipment at home ^3^	2.2 (0.7)	2.3 (0.7)	0.01*
Parent perceived importance of electronic media equipment in the bedroom at home ^3^	2 (0.8)	2.3 (0.8)	< 0.01*
Parent perceived importance of active play equipment at home ^3^	4.2 (0.8)	4 (0.8)	< 0.01*
** Rules, m (SD)**			
No. of outdoor safety rules	2 (1.1)	0.9 (1)	< 0.01*
No. of media rules	2.1 (0.8)	2.1 (0.8)	0.68
Maximum h/day of screen-time rule (% yes)	67%	50%	< 0.01*
No. of indoor rules	0.9 (0.8)	1.1 (0.8)	0.04*

^1^ 1 = strongly disagree; 5 = strongly agree

^2^ 1 = almost always—sedentary; 5 = almost always—PA

^3^ 1 = very unimportant; 5 = very important

^a^ Displayed for descriptive purposes only

**Table 2:** Descriptive statistics and differences for outcome variables between pre-COVID-19 and during COVID-19

	**Pre-COVID-19** **(2017–2018)**			**During COVID-19 (2020)**			
	**Mean (SD)**	**%**		**Mean (SD)**	**%**		***P***
% of time spent at home ^a^	-	46%		-	84%		< 0.01*
Time at home (h/day) ^a^	5.84 (1.4)	-		12.4 (6.6)	-		< 0.01*
**Home-based ActivPAL outcomes**			*n* = *96*			*n* = *95*	
Number of days of activPAL wear at home ^a^	5.38 (1.38)	-		6.29 (1.06)			< 0.01*
h/day of activPAL wear at home ^a^	6.29 (1.06)	-		10.30 (2.02)			< 0.01*
Min/h spent sitting, % of time at home*	39.6 (6.2)	66%		44.9 (6.1)	75%		< 0.01*
Min/h spent standing, % of time at home*	12.5 (4.3)	21%		7.5 (4.4)	13%		< 0.01*
Min/h spent stepping ^a^, % of time at home *	7.9 (2.9)	13%		4.4 (2.2)	7%		< 0.01*
Number of sitting breaks/h	7.2 (1.9)	-		5.3 (1.7)	-		< 0.01*
**Home-based ActiGraph outcomes**			*n* = 97			*n* = 98	
Number of days of ActiGraph wear at home ^a^	5.62 (1.33)	-		6.31 (1.11)			< 0.01*
h/day of ActiGraph wear at home ^a^	6.31 (1.11)	-		11.04 (1.36)			< 0.01*
Min/h spent in MVPA, % of time at home*	6.9 (2.4)	12%		4.5 (2.0)	8%		< 0.01*
Min/h spent in TPA, % of time at home*	21.6 (4.8)	36%		16.4 (4.3)	27%		< 0.01*

^*^% = proportion of time at home

^a^ Displayed for descriptive purposes only

Repeated linear regression analyses were conducted in R version 4.0.2 (http://cran.r-project.org) using the *stats* (version 4.2.0) and *lmtest (version* 0.9–38*)* packages. The unadjusted associations between the change in each physical and social environment variable and variables capturing the family situation during the first lockdown (home schooling, parents working from home, structure of the day (if home schooling) and the five home-based outcomes were examined using linear regression (Model 1). Model 2 adjusted for the following pre-determined covariates; age, sex, and BMI z-score of the child, home ownership, raw WIMD scores, season (pre-COVID-19), the number of siblings at home, dog ownership, and family situation.

A final statistical model (Model 3) was run for each of the five outcomes including all significant variables (*p* ≤ 0.10) from model 2 and adjustment variables, to determine independent associations between change in the physical and social environment factors and the child home-based outcomes. The significant results from this final model are presented in Table 3; the results from Model 1 and 2 and non-significant results from the final model are in supplementary material online. The summary scores (total number and the availability and accessibility scores) for each type of equipment were strongly correlated (r ≥ 0.60). The total number of equipment variables were more strongly related to the outcomes on average, therefore they were included in the final models [54].

**Table 3:** Associations between changes to the home environment and changes in the home-based behavioural outcomes

**Dependent variable**		**TPA **^**a**^	**MVPA **^**a**^	**Sitting **^**a**^	**Sitting breaks **^**a**^	**Standing **^**a**^
PA equipment quantity	**B (SE)**	–	–	-0.08 (0.05)	–	0.09 (0.03)
	**β**	–	–	-0.19	–	0.30
	***P***	–	–	0.09	–	< 0.01*
Media equipment quantity	**B (SE)**	–	–	–	-0.10 (0.05)	–
	**β**	–	–	–	-0.20	–
	***P***	–	–	–	0.05*	–
Child activity preferences	**B (SE)**	1.57 (0.53)	0.68 (0.22)	-1.89 (0.80)	–	0.71 (0.47)
	**β**	0.26	0.26	-0.25	–	0.14
	***P***	< 0.01*	< 0.01*	0.02*	–	0.14
Child social preferences	**B (SE)**	0.30 (0.32)	–	-1.02 (0.42)	0.22 (0.12)	0.54 (0.29)
	**β**	0.08	–	-0.23	0.14	0.18
	***P***	0.36	–	0.02*	0.08	0.06
Outdoor safety rules	**B (SE)**	–	–	–	–	-0.38 (0.28)
	**β**	–	–	–	–	-0.13
	***P***	–	–	–	–	0.19
Indoor play rules	**B (SE)**	–	–	0.62 (0.57)	–	–
	**β**	–	–	0.10	–	–
	***P***	–	–	0.28	–	–
Importance of electronic media equipment at home	**B (SE)**	–	–	1.03 (0.81)	–	-0.58 (0.51)
	**β**	–	–	0.14	–	-0.12
	***P***	–	–	0.21	–	0.26
Importance of active play equipment at home	**B (SE)**	–	–	–	0.14 (0.16)	–
	**β**	–	–	–	0.08	–
	***P***	–	–	–	0.41	–
Max h/day of screen-time (instated)	**B (SE)**	–	–	–	–	-0.09 (0.85)
	**β**	–	–	–	–	-0.02
	***P***	–	–	–	–	0.92
Max h/day of screen-time rule (removed)	**B (SE)**	–	–	–	–	1.87 (1.12)
	**β**	–	–	–	–	0.49
	***P***	–	–	–	–	0.10
Attending school (ref: No)						
*Sometimes*	**B (SE)**	-2.16 (0.78)	-0.73 (0.33)	–	–	–
	**β**	-0.49	-0.38	–	–	–
	***P***	0.01*	0.03*	–	–	–
Parent 1 working from home (ref: No)						
*Sometimes*	**B (SE)**	–	–	1.50 (1.78)	-0.61 (0.49)	–
	**β**	–	–	0.27	-0.31	–
	***P***	–	–	0.40	-0.22	–
*A few hours per day*	**B (SE)**	–	–	1.41 (1.84)	-0.17 (0.58)	–
	**β**	–	–	0.25	-0.09	–
	***P***	–	–	0.45	0.77	–
*Full time*	**B (SE)**	–	–	2.73 (1.45)	-0.76 (0.42)	–
	**β**	–	–	0.49	-0.39	–
	***P***	–	–	0.06	0.08	–
**R2 (adjusted R2):**		0.51 (0.40)	0.52 (0.42)	0.52 (0.37)	0.59 (0.49)	0.52 (0.37)

Final model including all significant variables from model 2 (*p* =  ≤ 0.10), adjusting for child BMI, age and sex, and season, number of siblings, home ownership, WIMD and dog ownership

^a^ Home-based behavioural outcomes

^*^ *p* ≤ 0.0

## Results

The descriptive statistics for COVID-19 variables and participants’ characteristics are presented in Tables 4 and 5. Children (50% girls) had a mean age of 10.2 ± 0.7 years and 12.8 ± 0.8 years before and during COVID-19, respectively. Participating parents were female (82%), owned their home (88%), had a university degree (60%), and lived in the highest SES location (56%). During COVID-19, the two parents were at home during the day (58%), with both parent 1 (usually the mother) [62%] and parent 2 (54%) working from home at least some of the time in the majority of families. Additionally, most children were not attending school (61%) and were home-schooled (89%).

**Table 4:** Participants’ characteristics

**Parent Characteristics**	**Pre-COVID-19**		**During COVID-19**	
	**(2017–2018)**		**2020**	
	Mean (SD) or %	*n*	Mean (SD) or %	*n*
Parent age	42 (5.6)	101	44.9 (6.7)	101
Parent sex (% Female)	86%	101	82%	101
Parental education ^a^		99		101
1 Some secondary high school	3%		6%	
2 Completed secondary high school	8%		9%	
3 Trade qualifications/apprenticeship	5%		5%	
4 Diploma/certificate	24%		20%	
5 University degree or higher	59%		60%	
**Child Characteristics**				
Child age	10.2 (0.7)	102	12.8 (0.8)	102
Child sex (% Girl)	50%	102	50%	102
Child BMI z-score	0.4 (1.1)	102	0.4 (1.6)	102
**Family Characteristics**				
Number of siblings	1.2 (0.8)	101	1.1 (0.8)	101
Number of people at home	4.2 (0.9)	101	4.2 (0.9)	101
Dog ownership [% yes]	36%	101	35%	101
Household		101		101
Single parent	15%		13%	
Two parent	85%		87%	
Other	-		-	
Home ownership		101		101
Rent	11%		12%	
Own	89%		88%	
SES (based on WIMD scores) ^a^		99		100
Low	13%		14%	
Medium	29%		30%	
High	58%		56%	

^a^ Displayed for descriptive purposes only

**Table 5:** Descriptive statistics for COVID-19 variables

Variables (*n* = 102)	%
Attending school [% yes]	40%
Home-schooling [% yes]	89%
Both parents at home [% yes]	58%
Parent 1 working from home (ref: No)	38%
Sometimes	18%
A few hours per day	10%
Full time	34%
Parent 2 working from home (ref: No)	14%
Sometimes	12%
A few hours per day	7%
Full time	35%
NA	32%

### Description of the pre-COVID-19 sample

Table 6 shows the key baseline descriptive characteristics pre-COVID-19 of the whole HomeSPACE project sample (*n* = 213), as well as the differences in these baseline characteristics between children that only participated pre-COVID-19 (*n* = 111) and children that participated in both pre-COVID-19 and during COVID-19 (*n* = 102). Children who did not participate during COVID-19 had higher BMI (*p* = 0.03) and spent more time sitting at home at baseline (*p* = 0.05). There were no other differences between children who participated during COVID-19, and those who did not.

**Table 6:** Characteristics of the whole sample of families participating in the HomeSPACE project at baseline (pre-COVID), and differences in key baseline characteristics between children that participated or not during COVID-19

	**Whole sample at baseline**		**Families participating during COVID-19**		**Families not participating during COVID-19**		
	***n***	**Mean (SD)**	***n***	**Mean (SD)**	***n***	**Mean (SD**	***P***
**Child characteristics**							
Child age (y)	213	10.2 (0.7)	102	10.2 (0.7)	111	10.2 (0.6)	0.76
Child sex (% Girl)	213	111, 52%	102	48, 47%	111	63, 59%	0.16
Child BMI z-score	212	0.6 (1.1)	102	0.4 (1.1)	110	0.8 (1.1)	0.03*
**Parent/family characteristics**							
Parent age (y)	206	41.6 (5.8)	101	41.9 (5.6)	105	41.2 (5.9)	0.38
Parent sex (% Female)	207	172, 83%	101	87, 86%	106	85, 80%	0.25
Parental education- University degree (*n*, %)	202	111, 55%	99	60, 61%	103	51, 50%	0.11
SES (based on WIMD scores) (*n*, %)	208		100		108		
Low		27, 13%		13, 13%		14, 13%	0.99
Medium		54, 26%		28, 28%		26, 24%	0.52
High		127, 61%		59, 59%		68, 63%	0.56
**Home-based behaviour outcomes**							
Min/h spent in TPA	195	21.4 (4.7)	97	21.5 (4.7)	98	21.3 (4.6)	0.73
Min/h spent in MVPA	195	6.7 (2.3)	97	6.8 (2.4)	98	6.5 (2.1)	0.37
Min/h in sitting	191	40.3 (5.8)	96	39.4 (6)	95	41.1 (5.5)	0.05*
Min/h in standing	191	12.2 (4.2)	96	12.6 (4.3)	95	11.9 (4)	0.25
Number of sitting breaks/h	191	6.9 (1.8)	96	7.1 (1.9)	95	6.7 (1.8)	0.17

Values are means (SD) unless otherwise indicated. P*: differences between children who participated or not during COVID-19 (independent *t* test for continuous variables or Chi squared test for categorical variables)

^*^ *p* ≤ 0.05

### Changes in the physical environment

The changes in the home physical environment from pre-COVID-19 to during COVID-19 are presented in Table 1. Households had a significantly larger amount of electronic games (*p* =  < 0.01), smart phones (*p* =  < 0.01), fitness trackers (*p* =  < 0.01), seated furniture (+ 1.5, *p* =  < 0.01), electronic media equipment overall (+ 1.2, *p* = 0.01) and in the primary child’s bedroom (+ 0.5, *p* =  < 0.01). Alternately, the number of books (*p* =  < 0.01) and PA equipment decreased (-1.1, *p* = 0.02) and subscriptions to streaming services increased (+ 16%, *p* = 0.01). There were no changes in the number of musical instruments or active video games at home.

### Changes in the social environment

The changes in the home social environment are reported in Table 1. Parents had implemented more rules relating to indoor play (+ 0.2, *p* = 0.04) and fewer regarding outdoor safety (-1.1, p =  < 0.01). Further, parents placed less importance on active play equipment (+ 0.2, *p* =  < 0.01) and more importance on electronic media equipment overall at home (+ 0.1, *p* = 0.01) and in the child’s bedroom (+ 0.3, *p* =  < 0.01), but fewer enforced a maximum h/day screen time rule (-17%, *p* =  < 0.01). In contrast, children’s preference for physical activities (-0.3, *p* =  < 0.01) and socialising with other family members at home decreased (-0.7, *p* =  < 0.01). Parents’ perceptions of the physical environment (outdoor space for play, indoor space for play, back outdoor supportiveness, front outdoor safety and front outdoor visibility), the number of media rules and their priorities relating to active play, media use, living space, and space for play did not change.

### Changes in sitting time, standing, the number of sitting breaks and TPA and MVPA

Changes in children’s time at home, sitting time, standing, the number of sitting breaks and TPA and MVPA at home are shown in Table 2. Children’s time at home increased (from 5.8 to 12.4 h/day, *p* =  < 0.01). Children’s home-based sitting increased (from 39.6 to 44.9 min/h, *p* =  < 0.01), whilst their home-based standing (from 12.5 to 7.5 min/h), TPA (from 21.6 to 16.4 min/h), MVPA (from 6.9 to 4.5 min/h) and the number of sitting breaks (from 7.2 to 5.3 min/h) all significantly decreased per hour (*p* < 0.01).

### Changes to the physical and social environment associated with changes in home-based TPA

Child preference for PA was positively associated with home-based TPA (*β* = 0.31, *p* =  < 0.01) (see supplementary material, Model 2, Table 5). Home-based TPA decreased in children who were attending school (*β* = -0.39, *p* = 0.04). In the final model, the positive association with child preference for PA (*β* = 0.26, *p* =  < 0.01), and the negative association with attending school remained significant (*β* = -0.49, *p* = 0.01) (Table 3).

### Changes to the physical and social environment associated with changes in home-based MVPA

Child preference for PA was positively associated with home-based MVPA (*β* = 0.27, *p* =  < 0.01), whilst attending school was negatively associated with home-based MVPA (*β* = -0.39, *p* = 0.04) (see supplementary material, Model 2, Table 6). In the final model, the positive association between MVPA and child preference for PA (*β* = 0.26, *p* =  < 0.01) and the negative association with attending school remained significant (*β* = -0.38, *p* = 0.03) (Table 3).

### Changes to the physical and social environment associated with changes in home-based sitting

The parent completing the questionnaire working full time at home was positively associated with home-based sitting (+ 3 min/h, *p* = 0.03). PA equipment was negatively associated with home-based sitting (*β* = -0.23, *p* = 0.03) (See supplementary material, Model 2, Table 1). Child preference for PA (*β* = -0.28, *p* =  < 0.01) and child preference for socialising at home (*β* = -0.29, *p* =  < 0.01) were negatively associated with sitting at home. The negative associations between child preference for socialising at home (*β* = -0.25, *p* = 0.02), and child preference for PA at home (*β* = -0.23, *p* = 0.02) remained significant in the final model (Table 3).

### Changes to the physical and social environment associated with changes in home-based sitting breaks

The number of media equipment items at home (*β* = -0.20, *p* = 0.05) and the perceived importance of active play equipment at home were negatively associated (*β* = -0.20, *p* = 0.03), whilst child preference for socialising at home was positively associated with sitting breaks at home (*β* = 0.17, *p* = 0.04) (See supplementary material, Model 2, Table 2). Only the negative association between the number of media equipment items (*β* = -0.20, *p* = 0.05) remained significant in the final model (Table 3).

### Changes to the physical and social environment associated with changes in home-based standing

The number of PA equipment (*β* = 0.30, *p* =  < 0.01), child preference for socialising at home (*β* = 0.23, *p* = 0.03), child preference for PA (*β* = 0.20, *p* = 0.05) and removing a screen-time limit (*β* = 0.71, *p* = 0.04) were all positively associated with standing at home (See supplementary material, Model 2, Table 4)). In the final model, the positive association with PA equipment remained (*β* = -0.30, *p* =  < 0.01) (Table 3).

## Discussion

The aims of this study were to: 1) examine changes in social and physical environmental factors at home and children’s home-based behavioural outcomes from pre-COVID-19 to during the first COVID-19 lockdown; 2) report associations between changes occurring at home and changes in children’s home-based behavioural outcomes as a result of the lockdown. This is the first study to explore changes in the home environment and their effects on PA and sitting at home during the COVID-19 pandemic. Children’s time at home increased significantly from 46% of waking time to 84% during the first COVID-19 lockdown, demonstrating the increased importance of the home environment to the regulation of movement behaviours during COVID-19. Not surprisingly, such significant time at home also led to several changes in the environment and children’s behaviour at home. Further, some changes in the home were also significantly associated with changes in children's behavioural outcomes.

Children’s PA, standing and sitting breaks at home declined during the first COVID-19 lockdown, while sitting increased compared with pre-COVID-19. The increase in sitting time is consistent with previous data reporting children’s sedentary time during COVID-19 [16, 19, 20, 55, 56]. The increase in sitting time partly reflects children engaging in school-work at home due to school closures [57]. However, one study showed that school related sedentary time only accounted for 90 min of the day during COVID-19 [16], suggesting that the increase in sitting time is more likely explained by greater time spent in sedentary pursuits such as TV viewing and video games as reported by other studies [16, 19]. Notably, there were also corresponding decreases in MVPA and TPA at home. In contrast, an increase in home-based PA has been reported in US children during the pandemic [16], although this study did not account for the proportional increase in time spent at home. While we found that total PA at home increased because overall time at home increased, relatively, children spent less time in PA at home during the lockdown restrictions. More time spent in inactivity and screen-time increases the risk of obesity and poor mental health in children, and can also negatively impact on academic performance [1, 58].

During the pandemic, both the physical and social environments in homes were more conducive to electronic media pursuits. The amount of media equipment increased by 10% in the home, and by 29% in children’s bedrooms from pre to during the first COVID-19 lockdown. The number of families subscribing to a movie-TV streaming service increased by 17%, and 17% fewer parents enforced a limit on screen-time. Parent’s also placed more importance on having electronic media equipment at home and in the child’s bedroom. The changes may, in part, reflect the increased prevalence of leisure screen-based sedentary pursuits among children and their families [16, 20, 55] and their families [19]. This is partly due to parents working from home and using electronic media equipment to keep their children entertained while they engaged in work tasks [59]. The greater accessibility of screen-based media during the pandemic [19] and its reinforcing nature [60] may also have had an impact, consistent with the Behavioural Change Theory (BCT) that accessibility and reinforcing value influence the choice to engage in types of behaviour and activities [61]. Although some families may have used remote and streaming services to engage in PA which would have contributed to increased screen usage, in a sample of American children aged 9–13, only 16.9% and 12.9%, participated in team sports and activity classes or lessons remotely, respectively [16]. Nevertheless, the changes are concerning, given screen-time, particularly TV viewing, is associated with unfavourable body composition, metabolic profiles, lower fitness and poor mental health outcomes in children [2] and adults [62]. In support of this, the increase in household media equipment was associated with a decrease in children’s sitting breaks. A greater amount of media equipment available for use at home may keep children entertained for longer, resulting in more prolonged periods of sitting and fewer sitting breaks. This is a significant problem, given sedentary time of a prolonged nature, is associated with less favourable body composition and metabolic profiles in children [58]. Whilst all other changes in the electronic media environment were not associated with behavioural outcomes, the observed changes may result in children adopting new behavioural habits of such high sedentary and screen time, and low levels of PA and sit to stand transitions that may be difficult to change when COVID-19 restrictions are lifted.

Home environments became less supportive of PA during the first lockdown, whereby the amount of PA equipment at home and the importance parents placed on it decreased. Children spent 79% of their time at home, and the lack of outdoor space [63] resulted in children spending much of this time indoors [64]. From a social family climate perspective parents’ view indoors as space for sedentary activities such as electronic media use and reading [65], reflected in the increase in the number of rules relating to indoor play in this study. Therefore, the decrease in PA equipment and the importance placed on it may be indicative of parents restricting its use. On the other hand, it could also be attributed to families spending more time in sedentary behaviours and less in PA during the first lockdown [19]. The decrease in PA equipment at home was also negatively associated with home-based standing during the first period of COVID-19 restrictions. The reason why the decrease in PA equipment did not affect TPA or MVPA, may be explained by a lack of space in most UK homes limiting children’s opportunities to be active while at home [63]. Active video games, throwing a ball back and forth, table tennis and trampolining are activities that require PA equipment, can feasibly occur in most homes and are usually performed standing [66]. Physical activity equipment at home has the potential to not only reduce sitting by increasing ambulatory movement, but it also serves to interrupt prolonged bouts of sitting. Given the limited opportunities to engage in MVPA at home, purchasing more PA equipment for the home could be a feasible strategy for replacing sitting time with light PA. Whilst there are cost implications to purchasing PA equipment, the weak association between ownership of PA equipment and income reported in other studies [67, 68], suggests it is not a major barrier.

Children’s preference for being active at home decreased during the first COVID-19 lockdown, compared with the period prior to the pandemic. Children’s preference for being physically active at home was also strongly positively associated with home-based TPA and MVPA, and negatively associated with sitting. These findings are in line with research that has shown a preference for being sedentary or physically active to be a strong predictor of children’s PA [5, 52] and screen-use [69]. Interestingly, children’s preference for interacting with other family members also decreased in the first lockdown. During lockdown, any conflicts between families’ members would have been exacerbated by reduced personal and social space [70], particularly in crowded households [71]. Children’s desire to socialise with family members was also negatively associated with sitting at home. As a result of this social preference, children may have spent more time alone in their bedrooms, which is associated with greater screen-based sedentary time [72, 73]. In addition, during a time when physical contact with friends was limited, increased time spent in bedrooms away from family members may result in heightened feelings of loneliness and depression [74]. Although, some children used social media and online gaming to keep in touch with friends during the pandemic [75], over use of these can be harmful [76]. In particular, excessive social media use has been associated with anxiety and loneliness during the pandemic [77, 78]. Therefore, strategies which encourage children to spend more screen-free time with their families could be important for their mental health and reducing their screen- use post COVID-19 [79].

The home situation (e.g., parents working from home, home schooling) during the COVID-19 restrictions has an important influence on children’s PA and sedentary time [80]. In this study, whether or not the child attended school was a strong predictor of behaviour which is in line with the ‘structured day hypothesis’ and recognises the importance of the structure of the school day and how this regulates health behaviours such as PA and sedentary behaviour [81]. Specifically, TPA and MVPA had the largest decrease in the group of children who attended school “sometimes” compared to the group who “did not attend at all.” For many families home schooling provided more flexible days, allowing more time for PA [80]. Due to increased opportunities to be active, children who were home-schooled may have been more physically active at home compared to those who attended school. Another possible explanation for this finding is that children returning to school coincided with the end of sports/activity class [82]. Children who engaged in sports once or more a week prior to the pandemic may have been doing this at home during the peak of lockdown, but outside the home as restrictions eased [16]. Whilst increased access to organised sport and activity classes could have led to a net increase in overall PA, the decreased PA at home remained a concern given PA would have been displaced with less desirable sedentary behaviours at home [58].

Methodological strengths of this study include the comprehensive nature of the HomeSPACE-I and II instruments used to capture the social and physical environments at home, the repeated measures study design and the use of device-based measures of sitting and PA as well as the home-based measure of behaviour. Moreover, a large number of confounding factors were controlled for in the models, which explained between 51 and 59% of the variance in the home-based behavioural outcomes, albeit baseline values accounted for a significant proportion of this. Additionally, to our knowledge this is the only study to assess the changes in the home environment as a result of the pandemic and the effects on children’s PA and sitting at home. Yet, the study is not without limitations. Firstly, from the pre-covid study sample, only 49% participated during the restrictions and these children had lower BMI and spent less time sitting at home at baseline. However, the extent to which this biased results is unclear. Families from high SES backgrounds were over-represented, however the proportion of low and medium SES families was higher than most previous studies [48, 83]. The study was also limited by the 2-year gap between the assessments. Children become more sedentary [84, 85] and less active as they get older [86, 87], therefore the changes observed in this study may, in part, be a result of age-related changes in behavioural habits rather than the restrictions in isolation. However, declines in MVPA (34% vs 14%), TPA (28% vs 22%) and sitting breaks at home (27% vs 26%) in this study were more pronounced compared with typical changes seen in children between 9–12 years [88, 89]. Whilst the increase in sitting is not as pronounced as increases reported elsewhere [85], a possible explanation for this is that we measured sitting at home whereas others have measured it across the entire day. Indeed, the change in sitting at home during the pandemic may be less pronounced than other behaviours, given sitting time at home relative to other locations was already high even before the pandemic [7, 11]. The extent to which the 2-year gap between measurements influenced results remains unclear, however based on comparisons with other studies, it is unlikely that the changes in this study are solely attributable to age-related changes in behavioural habits.

## Conclusion

The COVID-19 restrictions necessary to mitigate the spread of the virus required children to spend almost all their time at home during the first lockdown. The HomeSPACE study provides evidence that children’s PA, standing and sitting breaks at home decreased during the first lockdown, whilst sitting time increased. Homes also became less supportive of PA, and more conducive to electronic sedentary pursuits. These changes also impacted behaviour at home. An increase in PA equipment was positively associated with home-based standing, and an increase in media equipment was negatively associated with home-based sitting breaks. Decreased child preference for PA and socialising with family members also impacted behaviour. Specifically, children’s preference for PA was positively associated with TPA and MVPA, and negatively associated with sitting. Lastly, child preference for socialising with family members was negatively associated with sitting. The findings are concerning, given the health benefits of PA [90] and the association between sedentary time, particularly screen-based, and poor health outcomes [2]. To avoid the negative changes in the home and possibility of children’s behaviour becoming habituated, public health efforts are needed to reset and promote children’s PA, and discourage their sitting at home post-lockdown.

## Supplementary Information

**Additional file 1:** **Appendix 1.** Associations between changes to the home environment and changes in children’s home-based TPA. **Appendix 2.** Associations between changes to the home environment and changes in children’s home-based MVPA. **Appendix 3.** Associations between changes to the home environment and changes in children’s home-based sitting time. **Appendix 4.** Associations between changes to the home environment and changes in children’s home-based sitting breaks. **Appendix 5.** Associations between changes to the home environment and changes in children’s home-based standing.

## Data Availability

The datasets used and/or analysed are available from the corresponding author on reasonable request.

## Abbreviations

PA: Physical Activity
MVPA: Moderate-Vigorous Physical Activity
TPA: Total Physical Activity
BMI: Body Mass Index
WIMD: Welsh Index of Multiple Deprivation
SES: Socio-economic Status
SD: Standard Deviation
COVID-19: Coronavirus Disease 2019
UK: United Kingdom
SE: Standard Error
WHO: World Health Organisation
