# Correction: Homes became the “everything space” during COVID-19: impact of changes to the home environment on children’s physical activity and sitting

**DOI:** 10.1186/s12966-023-01409-1

**Published:** 2023-04-28

**Authors:** Michael P. R. Sheldrick, Nils J. Swindell, Amie B. Richards, Stuart J. Fairclough, Gareth Stratton

**Affiliations:** 1 Research Centre in Applied Sports, Technology, Exercise and Medicine (A-STEM), Swansea University, Swansea, SA1 8EN UK; 2 Movement Behaviours, Health and Wellbeing Research Group, Department of Sport & Physical Activity, Edge Hill University, St Helens Road, Ormskirk, Lancashire L39 4QP UK

**Correction: Int J Behav Nutr Phys Act 19, 134 (2022)**

**https://doi.org/10.1186/s12966-022-01346-5**

Following publication of the original article [1], the authors identified that the table reference numbers were incorrectly presented in the text, and Tables 4 and 5 were interchanged.

The original article [1] has been corrected.
