# Supplementary material for: Homes became the “everything space” during COVID-19: impact of changes to the home environment on children’s physical activity and sitting
Source: Int J Behav Nutr Phys Act. 2022 Oct 21;19:134. doi: 10.1186/s12966-022-01346-5 (PMC9587651; doi:10.1186/s12966-022-01346-5)
Supplement: Supplementary file 1 — Additional file 1: Appendix 1. Associations between changes to the home environment and changes in children’s home-based TPA. Appendix 2. Associations between changes to the home environment and changes in children’s home-based MVPA. Appendix 3. Associations between changes to the home environment and changes in children’s home-based sitting time. Appendix 4. Associations between changes to the home environment and changes in children’s home-based sitting breaks. Appendix 5. Associations between changes to the home environment and changes in children’s home-based standing. [file 12966_2022_1346_MOESM1_ESM.docx]

**Appendices**

**Appendix 1.** Associations between changes to the home environment and changes in children’s home-based TPA

|  | **Model 1** | | | | **Model 2** | | | | **Model 3** | | | |
| --- | --- | --- | --- | --- | --- | --- | --- | --- | --- | --- | --- | --- |
| **Physical home environment** | **B** | **(SE)** | **β** | ***P*** | **B** | **(SE)** | **β** | ***P*** | **B** | **(SE)** | **β** | ***P*** |
| Number of books | -0.26 | 0.48 | -0.05 | 0.60 | 0.09 | 0.48 | 0.02 | 0.85 |  |  |  |  |
| Number of electronic games | 0.25 | 0.46 | 0.06 | 0.59 | -0.11 | 0.50 | -0.03 | 0.82 |  |  |  |  |
| Number of smartphones | -0.15 | 0.97 | -0.03 | 0.87 | 0.41 | 1.03 | 0.09 | 0.69 |  |  |  |  |
| Number of fitness trackers | -1.02 | 0.51 | -0.21 | 0.05 | -0.73 | 0.49 | -0.15 | 0.14 |  |  |  |  |
| Number of PA equipment items | -0.04 | 0.04 | -0.13 | 0.23 | -0.03 | 0.03 | -0.08 | 0.42 |  |  |  |  |
| Number of media equipment item | 0.01 | 0.13 | 0.01 | 0.94 | 0.16 | 0.14 | 0.13 | 0.26 |  |  |  |  |
| Number of media equipment items in the bedroom | 0.31 | 0.30 | 0.12 | 0.30 | 0.16 | 0.30 | 0.06 | 0.59 |  |  |  |  |
| Number of seated furniture items | -0.06 | 0.08 | -0.08 | 0.46 | 0.02 | 0.07 | 0.02 | 0.83 |  |  |  |  |
| Signed up to streaming service | -0.95 | 1.16 | -0.22 | 0.42 | 0.05 | 1.19 | 0.01 | 0.97 |  |  |  |  |
| **Social home environment** |  |  |  |  |  |  |  |  |  |  |  |  |
| Child activity preferences | 1.26 | 0.61 | 0.21 | 0.04 | 1.85 | 0.54 | 0.31 | 0.00* | 1.57 | 0.53 | 0.26 | <0.01* |
| Child social preferences | 0.51 | 0.37 | 0.15 | 0.17 | 0.57 | 0.34 | 0.16 | 0.10* | 0.30 | 0.32 | 0.08 | 0.36 |
| Outdoor safety rules | -0.52 | 0.36 | -0.15 | 0.16 | -0.42 | 0.33 | -0.12 | 0.21 |  |  |  |  |
| Indoor play rules | 0.19 | 0.56 | 0.04 | 0.74 | -0.08 | 0.50 | -0.02 | 0.88 |  |  |  |  |
| Importance of electronic media equipment at home | -0.52 | 0.66 | -0.09 | 0.43 | -0.75 | 0.64 | -0.13 | 0.25 |  |  |  |  |
| Importance of electronic media equipment in bedroom | -0.53 | 0.49 | -0.11 | 0.28 | -0.53 | 0.53 | -0.11 | 0.33 |  |  |  |  |
| Importance of active play equipment at home | -0.28 | 0.30 | -0.09 | 0.36 | -0.28 | 0.31 | -0.09 | 0.37 |  |  |  |  |
| **Max h/day of screen-time rules (instated)** | -0.40 | 1.05 | -0.09 | 0.70 | -1.45 | 1.00 | -0.33 | 0.15 |  |  |  |  |
| **Max h/day of screen-time rule (removed)** | -2.01 | 1.50 | -0.46 | 0.18 | -1.02 | 1.41 | -0.23 | 0.47 |  |  |  |  |
| **COVID Home situation** |  |  |  |  |  |  |  |  |  |  |  |  |
| Attending school (ref: No) |  |  |  |  |  |  |  |  |  |  |  |  |
| *Sometimes* | -1.30 | 0.91 | -0.30 | 0.16 | -1.72 | 0.82 | -0.39 | 0.04* | -2.16 | 0.78 | -0.49 | 0.01* |
| Home schooling (ref: No) |  |  |  |  |  |  |  |  |  |  |  |  |
| *Yes* | 0.43 | 1.36 | 0.10 | 0.75 | -0.43 | 1.28 | -0.10 | 0.74 |  |  |  |  |
| Parent 1 working from home (ref: No) |  |  |  |  |  |  |  |  |  |  |  |  |
| *Sometimes* | -0.88 | 1.30 | -0.20 | 0.50 | -0.96 | 1.26 | -0.22 | 0.45 |  |  |  |  |
| *A few hours per day* | 0.06 | 1.58 | 0.01 | 0.97 | -0.35 | 1.47 | -0.08 | 0.81 |  |  |  |  |
| *Full time* | 0.46 | 1.10 | 0.11 | 0.68 | -0.08 | 1.09 | -0.02 | 0.94 |  |  |  |  |
| Both parents at home (ref: yes) |  |  |  |  |  |  |  |  |  |  |  |  |
| *No* | 1.02 | 0.95 | 0.23 | 0.29 | 1.07 | 0.92 | 0.24 | 0.25 |  |  |  |  |
| *N/A* | 0.66 | 2.61 | 0.15 | 0.80 | -0.03 | 2.93 | -0.01 | 0.99 |  |  |  |  |
|  |  |  |  |  |  |  |  |  | R ^2^ (adjusted R ^2^): 0.51 (0.40) | | | |

**Appendix 2.** Associations between changes to the home environment and changes in children’s home-based MVPA

|  | **Model 1** | | | | **Model 2** | | | | **Model 3** | | | |
| --- | --- | --- | --- | --- | --- | --- | --- | --- | --- | --- | --- | --- |
| **Physical home environment** | **B** | **(SE)** | **β** | ***P*** | **B** | **(SE)** | **β** | ***P*** | **B** | **(SE)** | **β** | ***P*** |
| Number of books | -0.09 | 0.21 | -0.04 | 0.67 | -0.08 | 0.2 | -0.04 | 0.70 |  |  |  |  |
| Number of electronic games | 0.19 | 0.2 | 0.1 | 0.34 | 0.09 | 0.21 | 0.05 | 0.66 |  |  |  |  |
| Number of smartphones | -0.36 | 0.41 | -0.19 | 0.38 | -0.08 | 0.43 | -0.04 | 0.85 |  |  |  |  |
| Number of fitness trackers | -0.27 | 0.22 | -0.13 | 0.23 | -0.32 | 0.2 | -0.15 | 0.12 |  |  |  |  |
| Number of PA equipment items | -0.01 | 0.02 | -0.04 | 0.72 | 0 | 0.01 | -0.02 | 0.86 |  |  |  |  |
| Number of media equipment item | -0.01 | 0.05 | -0.02 | 0.87 | 0.06 | 0.06 | 0.12 | 0.29 |  |  |  |  |
| Number of media equipment items in the bedroom | 0.17 | 0.13 | 0.14 | 0.19 | 0.16 | 0.12 | 0.14 | 0.18 |  |  |  |  |
| Number of seated furniture items | -0.03 | 0.03 | -0.11 | 0.3 | 0.01 | 0.03 | 0.03 | 0.74 |  |  |  |  |
| Signed up to streaming service | -0.46 | 0.5 | -0.24 | 0.35 | -0.06 | 0.5 | -0.03 | 0.90 |  |  |  |  |
| **Social home environment** |  |  |  |  |  |  |  |  |  |  |  |  |
| Child activity preferences | 0.59 | 0.27 | 0.22 | 0.03 | 0.7 | 0.23 | 0.27 | 0.00 | 0.68 | 0.22 | 0.26 | <0.01* |
| Child social preferences | 0.21 | 0.16 | 0.14 | 0.19 | 0.19 | 0.14 | 0.13 | 0.18 |  |  |  |  |
| Outdoor safety rules | -0.16 | 0.16 | -0.11 | 0.31 | -0.16 | 0.14 | -0.11 | 0.25 |  |  |  |  |
| Indoor play rules | -0.25 | 0.24 | -0.12 | 0.31 | -0.07 | 0.21 | -0.03 | 0.72 |  |  |  |  |
| Importance of electronic media equipment at home | -0.06 | 0.13 | -0.04 | 0.65 | -0.15 | 0.13 | -0.12 | 0.23 |  |  |  |  |
| Importance of electronic media equipment in bedroom | 0.12 | 0.29 | 0.05 | 0.66 | -0.24 | 0.27 | -0.1 | 0.38 |  |  |  |  |
| Importance of active play equipment at home | 0 | 0.21 | 0 | 0.99 | -0.04 | 0.23 | -0.02 | 0.87 |  |  |  |  |
| **Max h/day of screen-time rules (instated)** | -0.28 | 0.45 | -0.15 | 0.54 | -0.27 | 0.42 | -0.14 | 0.52 |  |  |  |  |
| **Max h/day of screen-time rule (removed)** | -1.13 | 0.65 | -0.59 | 0.08 | -0.91 | 0.58 | -0.48 | 0.12 |  |  |  |  |
| **COVID Home situation** |  |  |  |  |  |  |  |  |  |  |  |  |
| Attending school (ref: No) |  |  |  |  |  |  |  |  |  |  |  |  |
| *Sometimes* | -1.30 | 0.91 | -0.30 | 0.16 | -1.72 | 0.82 | -0.39 | 0.04* | -0.73 | 0.33 | -0.38 | 0.03* |
| Home schooling (ref: No) |  |  |  |  |  |  |  |  |  |  |  |  |
| *Yes* | 0.43 | 1.36 | 0.10 | 0.75 | -0.43 | 1.28 | -0.10 | 0.74 |  |  |  |  |
| Parent 1 working from home (ref: No) |  |  |  |  |  |  |  |  |  |  |  |  |
| *Sometimes* | -0.88 | 1.30 | -0.20 | 0.50 | 0.03 | 0.53 | 0.01 | 0.96 |  |  |  |  |
| *A few hours per day* | 0.06 | 1.58 | 0.01 | 0.97 | -0.32 | 0.62 | -0.17 | 0.61 |  |  |  |  |
| *Full time* | 0.46 | 1.10 | 0.11 | 0.68 | -0.1 | 0.46 | -0.05 | 0.82 |  |  |  |  |
| Both parents at home (ref: yes) |  |  |  |  |  |  |  |  |  |  |  |  |
| *No* | 1.02 | 0.95 | 0.23 | 0.29 | 0.5 | 0.38 | 0.26 | 0.20 |  |  |  |  |
| *N/A* | 0.66 | 2.61 | 0.15 | 0.80 | 0.39 | 1.23 | 0.2 | 0.75 |  |  |  |  |
|  |  |  |  |  |  |  |  |  | R ^2^ (adjusted R ^2^): 0.52 (0.42) | | | |

**Appendix 3.** Associations between changes to the home environment and changes in children’s home-based sitting time

|  | **Model 1** | | | | **Model 2** | | | | **Model 3** | | | |
| --- | --- | --- | --- | --- | --- | --- | --- | --- | --- | --- | --- | --- |
| **Physical home environment** | **B** | **(SE)** | **β** | ***P*** | **B** | **(SE)** | **β** | ***P*** | **B** | **(SE)** | **β** | ***P*** |
| Number of books | 0.39 | 0.62 | 0.07 | 0.53 | -0.24 | 0.64 | -0.04 | 0.70 |  |  |  |  |
| Number of electronic games | -0.34 | 0.59 | -0.06 | 0.57 | 0.64 | 0.67 | 0.11 | 0.34 |  |  |  |  |
| Number of smartphones | -0.63 | 1.22 | -0.11 | 0.6 | -0.85 | 1.36 | -0.15 | 0.53 |  |  |  |  |
| Number of fitness trackers | 0.59 | 0.64 | 0.09 | 0.36 | 0.88 | 0.64 | 0.14 | 0.17 |  |  |  |  |
| Number of PA equipment items | -0.06 | 0.04 | -0.15 | 0.15 | -0.1 | 0.04 | -0.23 | 0.03* | -0.08 | 0.05 | -0.19 | 0.09 |
| Number of media equipment item | 0.2 | 0.16 | 0.13 | 0.24 | -0.06 | 0.19 | -0.04 | 0.75 |  |  |  |  |
| Number of media equipment items in the bedroom | 0.17 | 0.4 | 0.05 | 0.67 | 0.38 | 0.42 | 0.11 | 0.37 |  |  |  |  |
| Number of seated furniture items | 0.02 | 0.1 | 0.02 | 0.87 | -0.09 | 0.09 | -0.1 | 0.37 |  |  |  |  |
| Signed up to streaming service | -0.21 | 1.46 | -0.04 | 0.89 | -1.56 | 1.62 | -0.28 | 0.34 |  |  |  |  |
| **Social home environment** |  |  |  |  |  |  |  |  |  |  |  |  |
| Child activity preferences | -1.96 | 0.77 | -0.25 | 0.01 | -2.12 | 0.72 | -0.28 | <0.01* | -1.89 | 0.80 | -0.25 | 0.02* |
| Child social preferences | -0.93 | 0.46 | -0.21 | 0.05 | -1.29 | 0.42 | -0.29 | <0.01* | -1.02 | 0.42 | -0.23 | 0.02* |
| Outdoor safety rules | 0.39 | 0.49 | 0.09 | 0.43 | 0.6 | 0.45 | 0.14 | 0.19 |  |  |  |  |
| Indoor play rules | 0.8 | 0.69 | 0.13 | 0.25 | 1.15 | 0.65 | 0.18 | 0.09* | 0.62 | 0.57 | 0.10 | 0.28 |
| Importance of electronic media equipment at home | 0.89 | 0.86 | 0.12 | 0.3 | 1.49 | 0.86 | 0.20 | 0.09* | 1.03 | 0.81 | 0.14 | 0.21 |
| Importance of electronic media equipment in bedroom | 0.24 | 0.64 | 0.04 | 0.71 | 0.23 | 0.72 | 0.04 | 0.74 |  |  |  |  |
| Importance of active play equipment at home | 0.12 | 0.39 | 0.03 | 0.75 | 0.48 | 0.41 | 0.12 | 0.25 |  |  |  |  |
| **Max h/day of screen-time rules (instated)** | -0.23 | 1.39 | -0.04 | 0.87 | 1.15 | 1.35 | 0.21 | 0.40 |  |  |  |  |
| **Max h/day of screen-time rule (removed)** | 0.13 | 1.95 | 0.02 | 0.95 | -1.49 | 1.90 | -0.27 | 0.44 |  |  |  |  |
| **COVID Home situation** |  |  |  |  |  |  |  |  |  |  |  |  |
| Attending school (ref: No) |  |  |  |  |  |  |  |  |  |  |  |  |
| *Sometimes* | 2.27 | 1.17 | 0.4 | 0.06 | 0.91 | 1.17 | 0.16 | 0.44 |  |  |  |  |
| Home schooling (ref: No) |  |  |  |  |  |  |  |  |  |  |  |  |
| *Yes* | -2.56 | 1.73 | -0.46 | 0.14 | -1.48 | 1.70 | -0.26 | 0.39 |  |  |  |  |
| Parent 1 working from home (ref: No) |  |  |  |  |  |  |  |  |  |  |  |  |
| *Sometimes* | -1.65 | 1.64 | -0.3 | 0.32 | 0.66 | 1.74 | 0.12 | 0.71 | 1.50 | 1.78 | 0.27 | 0.40 |
| *A few hours per day* | 1.52 | 2.1 | 0.27 | 0.47 | 1.65 | 1.94 | 0.29 | 0.40 | 1.41 | 1.84 | 0.25 | 0.45 |
| *Full time* | 1.34 | 1.42 | 0.24 | 0.35 | 3.15 | 1.46 | 0.56 | 0.03* | 2.73 | 1.45 | 0.49 | 0.06 |
| Both parents at home (ref: yes) |  |  |  |  |  |  |  |  |  |  |  |  |
| *No* | -1.47 | 1.22 | -0.26 | 0.23 | -1.86 | 1.25 | -0.33 | 0.14 |  |  |  |  |
| *N/A* | -5.38 | 3.29 | -0.96 | 0.11 | -5.39 | 3.87 | -0.96 | 0.17 |  |  |  |  |
|  |  |  |  |  |  |  |  |  | R ^2^ (adjusted R ^2^): 0.52 (0.37) | | | |

**Appendix 4.** Associations between changes to the home environment and changes in children’s home-based sitting breaks

|  | **Model 1** | | | | **Model 2** | | | | **Model 3** | | | |
| --- | --- | --- | --- | --- | --- | --- | --- | --- | --- | --- | --- | --- |
| **Physical home environment** | **B** | **(SE)** | **β** | ***P*** | **B** | **(SE)** | **β** | ***P*** | **B** | **(SE)** | **β** | ***P*** |
| Number of books | 0.15 | 0.22 | 0.07 | 0.48 | 0.05 | 0.19 | 0.03 | 0.79 |  |  |  |  |
| Number of electronic games | 0.13 | 0.21 | 0.06 | 0.54 | -0.05 | 0.19 | -0.02 | 0.81 |  |  |  |  |
| Number of smartphones | -0.23 | 0.43 | -0.12 | 0.6 | -0.05 | 0.41 | -0.03 | 0.90 |  |  |  |  |
| Number of fitness trackers | -0.33 | 0.22 | -0.15 | 0.14 | -0.29 | 0.19 | -0.13 | 0.13 |  |  |  |  |
| Number of PA equipment items | -0.01 | 0.02 | -0.08 | 0.46 | -0.01 | 0.01 | -0.1 | 0.31 |  |  |  |  |
| Number of media equipment item | -0.14 | 0.06 | -0.27 | 0.01 | -0.11 | 0.05 | -0.2 | 0.05* | -0.10 | 0.05 | -0.20 | 0.05* |
| Number of media equipment items in the bedroom | -0.08 | 0.14 | -0.06 | 0.59 | -0.07 | 0.13 | -0.06 | 0.59 |  |  |  |  |
| Number of seated furniture items | -0.01 | 0.03 | -0.04 | 0.72 | -0.01 | 0.03 | -0.02 | 0.82 |  |  |  |  |
| Signed up to streaming service | 0 | 0.51 | 0 | 1 | -0.11 | 0.49 | -0.06 | 0.82 |  |  |  |  |
| **Social home environment** |  |  |  |  |  |  |  |  |  |  |  |  |
| Child activity preferences | 0.29 | 0.27 | 0.11 | 0.29 | 0.35 | 0.23 | 0.13 | 0.13 |  |  |  |  |
| Child social preferences | 0.31 | 0.16 | 0.2 | 0.06 | 0.27 | 0.13 | 0.17 | 0.04* | 0.22 | 0.12 | 0.14 | 0.08 |
| Outdoor safety rules | -0.08 | 0.17 | -0.05 | 0.66 | -0.15 | 0.14 | -0.1 | 0.28 |  |  |  |  |
| Indoor play rules | 0.25 | 0.24 | 0.12 | 0.3 | -0.03 | 0.2 | -0.01 | 0.89 |  |  |  |  |
| Importance of electronic media equipment at home | -0.31 | 0.3 | -0.12 | 0.31 | -0.42 | 0.26 | -0.16 | 0.11 |  |  |  |  |
| Importance of electronic media equipment in bedroom | -0.2 | 0.22 | -0.1 | 0.35 | 0.07 | 0.22 | 0.03 | 0.76 |  |  |  |  |
| Importance of active play equipment at home | -0.17 | 0.13 | -0.12 | 0.21 | -0.27 | 0.12 | -0.20 | 0.03* | 0.14 | 0.16 | 0.08 | 0.41 |
| **Max h/day of screen-time rules (instated)** | -0.1 | 0.48 | -0.05 | 0.84 | -0.35 | 0.40 | -0.18 | 0.38 |  |  |  |  |
| **Max h/day of screen-time rule (removed)** | 0.2 | 0.67 | 0.1 | 0.76 | -0.18 | 0.56 | -0.09 | 0.75 |  |  |  |  |
| **COVID Home situation** |  |  |  |  |  |  |  |  |  |  |  |  |
| Attending school (ref: No) |  |  |  |  |  |  |  |  |  |  |  |  |
| *Sometimes* | -0.60 | 0.41 | -0.31 | 0.15 | -0.28 | 0.33 | -0.15 | 0.40 |  |  |  |  |
| Home schooling (ref: No) |  |  |  |  |  |  |  |  |  |  |  |  |
| *Yes* | 0.40 | 0.61 | 0.21 | 0.51 | -0.23 | 0.50 | -0.12 | 0.64 |  |  |  |  |
| Parent 1 working from home (ref: No) |  |  |  |  |  |  |  |  |  |  |  |  |
| *Sometimes* | -0.73 | 0.57 | -0.37 | 0.21 | -0.59 | 0.50 | -0.30 | 0.24 | -0.61 | 0.49 | -0.31 | -0.22 |
| *A few hours per day* | -1.09 | 0.73 | -0.55 | 0.14 | -0.44 | 0.58 | -0.23 | 0.45 | -0.17 | 0.58 | -0.09 | 0.77 |
| *Full time* | -0.94 | 0.49 | -0.48 | 0.06 | -0.78 | 0.43 | -0.40 | 0.08* | -0.76 | 0.42 | -0.39 | 0.08 |
| Both parents at home (ref: yes) |  |  |  |  |  |  |  |  |  |  |  |  |
| *No* | 0.59 | 0.43 | 0.30 | 0.17 | 0.35 | 0.38 | 0.18 | 0.35 |  |  |  |  |
| *N/A* | 1.70 | 1.15 | 0.87 | 0.14 | 0.38 | 1.15 | 0.19 | 0.74 |  |  |  |  |
|  |  |  |  |  |  |  |  |  | R ^2^ (adjusted R^2^): 0.59 (0.49) | | | |

**Appendix 5.** Associations between changes to the home environment and changes in children’s home-based standing

|  | **Model 1** | | | | **Model 2** | | | | **Model 3** | | | |
| --- | --- | --- | --- | --- | --- | --- | --- | --- | --- | --- | --- | --- |
| **Physical home environment** | **B** | **(SE)** | **β** | ***P*** | **B** | **(SE)** | **β** | ***P*** | **B** | **(SE)** | **β** | ***P*** |
| Number of books | -0.27 | 0.42 | -0.07 | 0.52 | 0.07 | 0.45 | 0.02 | 0.88 |  |  |  |  |
| Number of electronic games | 0.36 | 0.4 | 0.09 | 0.37 | 0.02 | 0.47 | 0.01 | 0.97 |  |  |  |  |
| Number of smartphones | 0.18 | 0.83 | 0.05 | 0.83 | 0.65 | 0.96 | 0.17 | 0.50 |  |  |  |  |
| Number of fitness trackers | -0.2 | 0.44 | -0.05 | 0.65 | -0.30 | 0.46 | -0.07 | 0.51 |  |  |  |  |
| Number of PA equipment items | 0.08 | 0.03 | 0.27 | 0.01 | 0.10 | 0.03 | 0.34 | <0.01* | 0.09 | 0.03 | 0.30 | <0.01* |
| Number of media equipment item | -0.2 | 0.11 | -0.2 | 0.07 | -0.15 | 0.13 | -0.14 | 0.27 |  |  |  |  |
| Number of media equipment items in the bedroom | -0.18 | 0.27 | -0.08 | 0.52 | -0.40 | 0.29 | -0.17 | 0.17 |  |  |  |  |
| Number of seated furniture items | 0.06 | 0.07 | 0.09 | 0.41 | 0.10 | 0.07 | 0.17 | 0.12 |  |  |  |  |
| Signed up to streaming service | -0.7 | 0.99 | -0.18 | 0.48 | -0.17 | 1.16 | -0.04 | 0.89 |  |  |  |  |
| **Social home environment** |  |  |  |  |  |  |  |  |  |  |  |  |
| Child activity preferences | 0.81 | 0.54 | 0.15 | 0.14 | 1.02 | 0.52 | 0.20 | 0.05* | 0.71 | 0.47 | 0.14 | 0.14 |
| Child social preferences | 0.36 | 0.32 | 0.12 | 0.26 | 0.69 | 0.30 | 0.23 | 0.03* | 0.54 | 0.29 | 0.18 | 0.06 |
| Outdoor safety rules | -0.33 | 0.33 | -0.11 | 0.33 | -0.53 | 0.32 | -0.18 | 0.10* | -0.38 | 0.28 | -0.13 | 0.19 |
| Indoor play rules | -0.11 | 0.44 | -0.03 | 0.81 | -0.37 | 0.46 | -0.09 | 0.42 |  |  |  |  |
| Importance of electronic media equipment at home | -0.82 | 0.58 | -0.16 | 0.16 | -1.02 | 0.61 | -0.2 | 0.10* | -0.58 | 0.51 | -0.12 | 0.26 |
| Importance of electronic media equipment in bedroom | -0.38 | 0.43 | -0.09 | 0.38 | -0.42 | 0.50 | -0.1 | 0.41 |  |  |  |  |
| Importance of active play equipment at home | 0.75 | 0.53 | 0.15 | 0.16 | 0.64 | 0.54 | 0.13 | 0.24 |  |  |  |  |
| **Max h/day of screen-time rules (instated)** | 1.26 | 0.93 | 0.33 | 0.18 | -0.10 | 0.94 | -0.03 | 0.91 | -0.09 | 0.85 | -0.02 | 0.92 |
| **Max h/day of screen-time rule (removed)** | 1.32 | 1.31 | 0.35 | 0.32 | 2.71 | 1.3 | 0.71 | 0.04* | 1.87 | 1.12 | 0.49 | 0.10 |
| **COVID Home situation** |  |  |  |  |  |  |  |  |  |  |  |  |
| Attending school (ref: No) |  |  |  |  |  |  |  |  |  |  |  |  |
| *Sometimes* | -1.46 | 0.80 | -0.38 | 0.07 | -0.30 | 0.82 | -0.08 | 0.71 |  |  |  |  |
| Home schooling (ref: No) |  |  |  |  |  |  |  |  |  |  |  |  |
| *Yes* | 1.68 | 1.17 | 0.44 | 0.16 | 1.19 | 1.17 | 0.31 | 0.31 |  |  |  |  |
| Parent 1 working from home (ref: No) |  |  |  |  |  |  |  |  |  |  |  |  |
| *Sometimes* | 1.59 | 1.12 | 0.42 | 0.16 | 0.42 | 1.24 | 0.11 | 0.73 |  |  |  |  |
| *A few hours per day* | -0.61 | 1.43 | -0.16 | 0.67 | -0.78 | 1.36 | -0.20 | 0.57 |  |  |  |  |
| *Full time* | -0.41 | 0.96 | -0.11 | 0.67 | -1.26 | 1.03 | -0.33 | 0.22 |  |  |  |  |
| Both parents at home (ref: yes) |  |  |  |  |  |  |  |  |  |  |  |  |
| *No* | 0.78 | 0.84 | 0.20 | 0.35 | 1.19 | 0.87 | 0.31 | 0.18 |  |  |  |  |
| *N/A* | 3.25 | 2.25 | 0.85 | 0.15 | 2.65 | 2.69 | 0.70 | 0.33 |  |  |  |  |
|  |  |  |  |  |  |  |  |  | R ^2^ (adjusted R ^2^): 0.52 (0.37) | | | |
